# Efficient and Spectrally Stable Blue Perovskite Light‐Emitting Diodes Employing a Cationic π‐Conjugated Polymer

**DOI:** 10.1002/adma.202103640

**Published:** 2021-09-24

**Authors:** Shuai Yuan, Lin‐Song Cui, Linjie Dai, Yun Liu, Qing‐Wei Liu, Yu‐Qi Sun, Florian Auras, Miguel Anaya, Xiaopeng Zheng, Edoardo Ruggeri, You‐Jun Yu, Yang‐Kun Qu, Mojtaba Abdi‐Jalebi, Osman M. Bakr, Zhao‐Kui Wang, Samuel D. Stranks, Neil C. Greenham, Liang‐Sheng Liao, Richard H. Friend

**Affiliations:** ^1^ Jiangsu Key Laboratory for Carbon‐Based Functional Materials & Devices Institute of Functional Nano & Soft Materials (FUNSOM) Soochow University Suzhou Jiangsu 215123 China; ^2^ Department of Polymer Science and Engineering University of Science and Technology of China Hefei Anhui 230026 China; ^3^ Cavendish Laboratory University of Cambridge JJ Thomson Avenue Cambridge CB3 0HE UK; ^4^ Division of Physical Sciences and Engineering King Abdullah University of Science and Technology (KAUST) Thuwal 23955‐6900 Kingdom of Saudi Arabia

**Keywords:** blue emissions, high efficiencies, ion migrations, perovskite light‐emitting diodes, stabilities

## Abstract

Metal halide perovskite semiconductors have demonstrated remarkable potentials in solution‐processed blue light‐emitting diodes (LEDs). However, the unsatisfied efficiency and spectral stability responsible for trap‐mediated non‐radiative losses and halide phase segregation remain the primary unsolved challenges for blue perovskite LEDs. In this study, it is reported that a fluorene‐based π‐conjugated cationic polymer can be blended with the perovskite semiconductor to control film formation and optoelectronic properties. As a result, sky‐blue and true‐blue perovskite LEDs with Commission Internationale de l'Eclairage coordinates of (0.08, 0.22) and (0.12, 0.13) at the record external quantum efficiencies of 11.2% and 8.0% were achieved. In addition, the mixed halide perovskites with the conjugated cationic polymer exhibit excellent spectral stability under external bias. This result illustrates that π‐conjugated cationic polymers have a great potential to realize efficient blue mixed‐halide perovskite LEDs with stable electroluminescence.

## Graphical abstract

A fluorene‐based π‐conjugated cationic polymer as a multifunctional passivator to suppress non‐radiative processes, improve charge transport properties, and inhibit ion migration for blue mixed‐halide perovskite semiconductors, is reported. As a result, efficient and spectrally stable blue perovskite light‐emitting diodes with emission wavelengths from 485 to 458 nm are achieved.

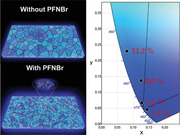

## 1 Introduction

Light‐emitting diodes (LEDs) based on perovskite materials have received strong research interest because of their high luminescence efficiency, tunable light emission, and good color purity.^[^ ^1^, ^2^, ^3^, ^4^ ^]^ External quantum efficiencies (EQEs) of over 20% have been achieved for near‐infrared, red, and green perovskite LEDs,^[^ ^5^, ^6^, ^7^, ^8^, ^9^ ^]^ while the performance of film‐based true‐blue perovskite LEDs (with emission <480 nm) far lags behind their counterparts emitting longer wavelength.^[^ ^10^, ^11^, ^12^, ^13^, ^14^, ^15^, ^16^, ^17^, ^18^ ^]^

Mixed‐halide Cl/Br perovskites afford the most facile way to achieve emission in the blue region. However, as these systems usually suffer from severe trap‐mediated non‐radiative losses, the photoluminescence quantum yield (PLQY) of thin films is relatively low (<40%), limiting their ultimate LED efficiency.^[^ ^19^, ^20^, ^21^, ^22^, ^23^ ^]^ Furthermore, due to the ionic nature of perovskite materials, the migration of halide ions is usually observed in mixed halide perovskites under external stimuli (electric field, light radiation, and thermal heating), resulting in shifted emission spectra and material decomposition.^[^ ^14^, ^15^, ^24^ ^]^ Moreover, the migration of the halide ions enables phase segregation, adding another obstacle for the development of high‐performance and operationally stable mixed‐halide perovskite LEDs.^[^ ^25^, ^26^, ^27^, ^28^, ^29^, ^30^ ^]^ With this in mind, breakthroughs have been made in mixed‐halide blue perovskite LEDs. Zhong and coworkers have successfully developed a dual‐ligand strategy to precisely control the dimension distribution for efficient blue mixed‐halide perovskite LEDs with a EQE of 8.8% at the emission wavelength of 473 nm.^[^ ^31^ ^]^ Gao's group has recently demonstrated spectrally stable mixed halide blue and deep‐blue perovskite LEDs through a vapor‐assisted crystallization technique.^[^ ^11^ ^]^ Albeit the significant progress in mixed‐halide blue perovskite LEDs, the trade‐offs among PLQY, spectral stability, and charge carrier transport in the mixed‐halide blue perovskite semiconductors are still a fundamental dilemma for further improvement in the corresponding device efficiencies and stability. Here, we demonstrate that incorporating the cationic π‐conjugated polymer poly [(9,9‐bis(3′‐((*N*,*N*‐dimethyl)‐*N*‐ethylammonium)‐propyl)‐2,7‐fluorene)‐*alt*‐2,7‐(9,9‐dioctylfluorene)] dibromide (PFNBr) can improve both the morphological and optoelectronic properties of mixed Cl/Br quasi‐2D perovskite films, resulting in efficient and stable blue LEDs. This improvement in the device performance with respect to previous reports is based on a substantially increased PLQY of ≈82%, which originates from a drastic reduction in the trap density in the PFNBr‐containing perovskite films. We speculate that the charged quaternary ammonium and bromide in PFNBr have the capability to passivate negatively and positively charged ionic defects at grain boundary of perovskite.^[^ ^29^, ^32^ ^]^ On the other hand, the quaternary ammonium cation group and bromide anion could also mitigate the migration of halide ions through a weak electrostatic interaction and grain boundary healing process,^[^ ^30^, ^33^, ^34^ ^]^ resulting in considerable improvement in the spectral stability of both the thin films and devices. In contrast with the insulating polymer additives generally employed in the field to modulate perovskite film,^[^ ^7^, ^35^ ^]^ the incorporation of PFNBr with semiconductor properties could improve the optical properties of the perovskite emitters without any sacrifice of their electrical conductivity. This is another potential reason for the improvement of spectral and device stability.

It is clear from the above discussion that the passivation mechanisms of the cationic π‐conjugated polymer in our study are different from other reported neutral π‐conjugated polymers used as Lewis bases for the coordination of Pb^2+^ ions.^[^ ^36^, ^37^ ^]^ As a result, our strategy is not only beneficial to improve the PLQY and charge carrier transport of the perovskite semiconductors, but also favorable for the spectral stability of both the perovskite thin films and devices. Therefore, the cationic π‐conjugated conductive polymer passivation approach reported here introduces a generic concept for the integral improvement of mixed‐halide blue perovskite LEDs.

## 2 Results and Discussion

**Figure 1:**
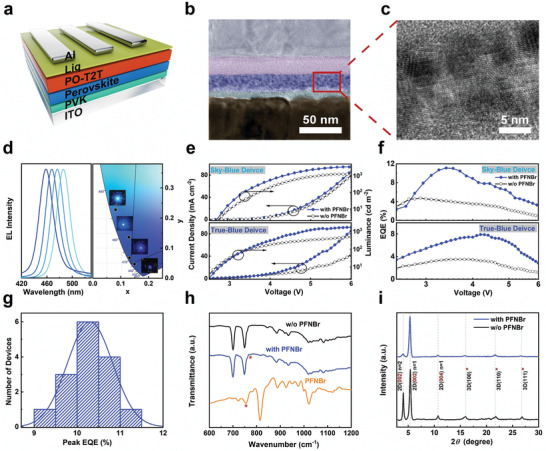
Perovskite LED architecture, performance, and perovskite film characteristics. a) Device architecture. b) TEM cross‐sectional image for the sky‐blue perovskite LED with PFNBr modification. c) A zoomed‐in TEM image showing the crystal structure of the PFNBr‐modified sky‐blue perovskite film. Since the quasi‐2D perovskite crystallites are randomly oriented, their layered structure is not visible in this projection. d) The electroluminescence (EL) spectra (left) and the corresponding CIE coordinates (right) of sky‐blue, true‐blue, and deep‐blue perovskite LEDs driven at voltage of 3.5 V. The inset shows the photograph for the sky‐blue, true‐blue, and deep‐blue working devices e) *J*–*V*–*L* curves of the sky‐blue (upper) and true‐blue perovskite LEDs with and without PFNBr modification. f) EQE characteristics of the sky‐blue (upper) and true‐blue (bellow) perovskite LEDs with and without PFNBr modification. g) Histogram of peak EQEs measured from sky‐blue perovskite LEDs with PFNBr modification. h) FTIR spectra of the sky‐blue perovskite films without (black) and with PFNBr modification (blue), and the pure PFNBr film (yellow). i) GIXRD patterns of the sky‐blue perovskite films with and without the PFNBr. The reflections at low angles correspond to the layered PEA_2_PbCl*_x_* Br_(4−_ *_x_*_)_ (*n* = 1) and PEA_2_CsPb_2_Cl*_y_* Br_(7−_ *_y_*_)_ (*n* = 2) phases. PEA = phenylethylammonium. *For simplicity, the bulk 3D CsPbCl*_z_* Br_(3−_ *_z_*_)_ phase is indexed in a cubic symmetry system. The actual unit cell might slightly deviate from this approximation.

We fabricated perovskite LEDs with a device architecture as follows: indium tin oxide (ITO, 120 nm) coated substrate/poly(9‐vinylcarbazole) (PVK, 8 nm)/perovskite (15 nm)/((1,3,5‐triazine‐2,4,6‐triyl) tris(benzene‐3,1‐diyl)) tris(diphenylphosphine oxide) (PO‐T2T, 25 nm)/ 8‐hydroxyquinolatolithium (Liq, 2 nm)/Al (100 nm) (**Figure** **1**a). The emitting film consists of blue‐emitting Cl/Br quasi‐2D perovskite materials prepared via spin‐coating from the precursor solutions in dimethyl sulfoxide (DMSO) containing phenylethylammonium bromide (PEABr), cesium bromide (CsBr), and (lead(II) bromide [PbBr_2_] + lead(II) chloride [PbCl_2_]) at a molar ratio of 0.8:1.0:0.95, as well as the PFNBr additive with an optimized concentration of 0.5 mg mL^−1^. Figure S1, Supporting Information, presents the molecular structure, absorption, and photoluminescence (PL) spectra of PFNBr. The Br/Cl mixed halide perovskite emitters provide wide‐range tunable emission spectra from sky‐blue to deep‐blue along with the increasing chloride concentration in the precursors (Figure S2, Supporting Information). Details about the film fabrication and precursor recipes could be found in the Experimental Section. With modification of PFNBr, the devices reveal dramatically enhanced performance. For sky‐blue emission, the control device without PFNBr modification shows a maximum luminance of 1250 cd m^−2^ and an EQE of 4.6%, whereas the PFNBr‐containing device has significantly increased EQE and maximum luminance of 11.2% and 3377 cd m^−2^, as shown in Figure 1e,f. The devices exhibit electroluminescence (EL) peak at 485 nm with the Commission Internationale de l'Eclairage (CIE) coordinates of (0.08, 0.22) (Figure 1d). The angular dependence of the luminous intensity is well fitted by a Lambertian distribution (Figure S3, Supporting Information). For true‐blue emission, the PFNBr‐containing device displays the maximum luminance and peak EQE as high as 1660 cd m^−2^ and 8.0% with the EL peak at 476 nm and the CIE coordinates of (0.12, 0.13), which are more than twofold higher than that of device without PFNBr modification (Figure 1e,f). This performance is, to the best of our knowledge, among the best results for the true‐blue perovskite LEDs to date (Table S1, Supporting Information). Moreover, we further modulated the emission spectrum of perovskites to deep‐blue region (Figures S4 and S5, Supporting Information), the peak EQEs of PFNBr‐containing devices are 3.2% and 1.9% with the EL peaks at 468 and 458 nm, respectively. It is notable that all devices with PFNBr treatment exhibit superior performance compared to the untreated devices. The turn‐on voltages of all devices are summarized in Table S2, Supporting Information. The EL spectra and the corresponding CIE coordinates of the devices with PFNBr modification are listed in Figure 1d. Furthermore, PFNBr‐containing devices show reasonable reproducibility. Taking sky‐blue devices as an example, the statistics of 15 devices prepared in three different batches exhibit an average peak EQE of ≈10.3% with a relative standard deviation of ≈5% (Figure 1g). To understand the underlying reasons for the improvement of device performance by adding PFNBr additive, we carried out a range of film characterizations as follow by using sky‐blue perovskite film as an example.

We first inspected the cross‐sectional transmission electron microscopy (TEM) (Figure 1b) and scanning electron microscopy (SEM) (Figure S6, Supporting Information) images; the PFNBr‐modified perovskite film sandwiched between the hole and electron transporting layers is homogeneous and pinhole‐free. The high‐resolution TEM (HR‐TEM) imaging (Figure 1c) reveals that the perovskite film is comprised of highly crystalline spherical‐shaped grains with a domain size of about 5 nm.

To further understand the mechanism behind the superior performance of the PFNBr treated device, we investigated effects of PFNBr on the formation of the Cl/Br quasi‐2D perovskite film and how the different molecular species interact. We performed dynamic light scattering experiments to unravel the dispersive properties of perovskite precursor solution (Figure S7, Supporting Information). The results suggest that the polymer reduces the size of the initially formed colloidal clusters and effectively inhibits the growth of larger aggregates. We attribute this to the interaction of the charged alkylammonium moieties of the polymer with the perovskite seeds. The size different between colloidal clusters in precursor solution and grains in the terminal perovskite films could be attributed to volume shrinkage during film formation.^[^ ^38^ ^]^ The interaction between the PFNBr and the perovskite is further confirmed by Fourier transform infrared spectroscopy (FTIR, Figure 1h). The absorption band of the PFNBr symmetric C—N vibration shifts from 756 to 773 cm^−1^ when mixed with the perovskite (Figure S8, Supporting Information), which can be largely attributed to a weak electrostatic interaction or ionic bonding between PFNBr and the perovskite surface.^[^ ^28^, ^29^, ^30^, ^33^, ^34^ ^]^ As expected from the suppressed aggregation in the precursor solution, the polymer additive also has a profound effect on the film formation and morphology, leading to more uniform and significantly smoother perovskite layers (Figure S9, Supporting Information). As revealed via grazing incidence X‐ray diffraction (GIXRD), these films are polycrystalline mixtures of the quasi‐2D PEA_2_PbCl*_x_* Br_(4−_ *_x_*_)_ (*n* = 1) and PEA_2_CsPb_2_Cl*_y_* Br_(7−_ *_y_*_)_ (*n* = 2) phases, where *n* is the number of lead halide layers, and the bulk 3D CsPbCl*_z_* Br_(3−_ *_z_*_)_ phases. Interestingly, the ratio between the *n* = 1, *n* = 2 and the 3D phases is shifted toward the single‐layer (*n* = 1) 2D perovskite when the PFNBr is involved (Figure 1i). It is important to note that the polymer with larger molecular size could not be incorporated into perovskite lattice as reflected by GIXRD. Further evidence from TEM image confirms that the polymer is located at grain boundary of perovskite (Figure S10, Supporting Information). Moreover, the average crystal domain sizes, calculated from the peak broadening of the 2D and 3D phases reflections, are drastically reduced (Table S3, Supporting Information). In particular, the 3D domains decreased from 14 to 8 nm, which is consistent with the HR‐TEM micrograph shown in Figure 1c. As result, the exciton binding energy of PFNBr‐modified perovskite increases by 50% with a value of 141.6 meV. (Figure S11, Supporting Information).

**Figure 2:**
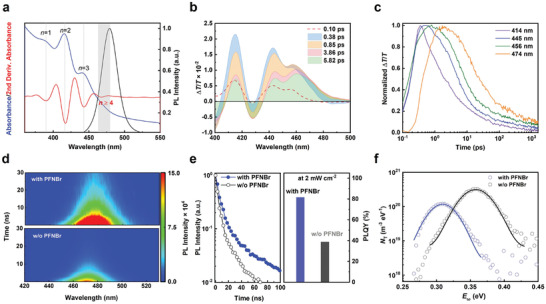
Photophysical properties and analysis of the trap densities of the perovskite films. a) UV–vis absorption (blue) and steady‐state PL emission (black) spectra of the PFNBr‐modified sky‐blue perovskite film. The red line is the second derivative of the absorption spectrum. The absorption features of the low‐dimensional phases with different numbers of perovskite layers are indicated. b) Transient absorption spectra of PFNBr‐modified sky‐blue perovskite film at different times. c) The corresponding decay kinetics at different wavelengths. d) Time‐resolved PL measurements of the sky‐blue perovskite films with and without PFNBr. e) Transient PL decay curves (left) and PLQEs of the sky‐blue perovskite films with and without PFNBr modification. f) Trap state distribution of the sky‐blue perovskite films with and without PFNBr modification measured at room temperature.

We carried out transient absorption spectroscopy to gain insight into the nature of the emissive species in the PFNBr‐containing quasi‐2D perovskite. As shown in **Figure** **2**a, three distinctive ground state bleach (GSB) peaks at 414, 445, and 456 nm are observed in the sky‐blue perovskite film. According to the second derivative of the steady‐state absorption in Figure 2b, the first two sharp bleaches agree well with the quasi‐2D perovskite absorption features of the bilayer (*n* = 2) and trilayer (*n* = 3) phases. In contrast, the third broad bleach feature is attributed to the spectrally overlapped signals of the *n* ≥ 4 components. The boundary between this band edge bleach feature and the bandgap renormalization feature at the early times (0.1 ps) determines the bandgap of the quasi‐3D components with *n* ≥ 4 to be at 474 nm, which corresponds to the emissive species as shown in Figure 2a. Figure 2c shows the dynamic evolution of the three bleach bands (414, 445, and 456 nm) and the band edge of the emissive species (474 nm). The band edge signal rises rapidly and then decays after 2 ps, whereas the 414 and 445 nm GSB signals decay quickly in 2ps, indicating an energy funneling process of excitations from the quasi‐2D (*n* = 2, 3) to quasi‐3D domains (*n* ≥ 4), on which they then undergo radiative recombination. A more detailed discussion can be found in Figure S12, Supporting Information.

The perovskite film with PFNBr modification exhibits stronger luminescence at a 0.2 mW cm^−2^ excitation intensity (Figure 2d). Time‐resolved PL measurements under the same power intensity further reveal an increased average PL lifetime of the sky‐blue quasi‐2D perovskite film from 13 to 26 ns after passivation by PFNBr (Figure 2e). The longer PL lifetime indicates reduction of non‐radiative recombination channels in the PFNBr‐modified film, leading to PLQY of ≈82%, which is more than twice the PLQY of the reference film of ≈39% (Figure 2e and Figure S13, Supporting Information). This enhancement in the PL characteristics of the passivated samples correlates with an important decrease in the trap density as shown by the capacitance versus frequency plot (Figure 2f and Figure S14, Supporting Information). The peak trap density of the PFNBr‐modified film decreased from 3.3 × 10^20^ to 1.4 × 10^20^ m^3^ eV^−1^ compared to the film without PFNBr modification, while the corresponding trap state distribution also became narrower and shallower in the PFNBr‐modified film. It can be explained that the charged quaternary ammonium and bromide in PFNBr are able to passivate negatively and positively charged ionic defects at grain boundary of perovskite responsible for deep level traps.^[^ ^29^, ^30^, ^34^ ^]^ The improved optoelectronic properties of the quasi‐2D system are responsible for enhancing EL efficiencies in the corresponding perovskite LEDs.

**Figure 3:**
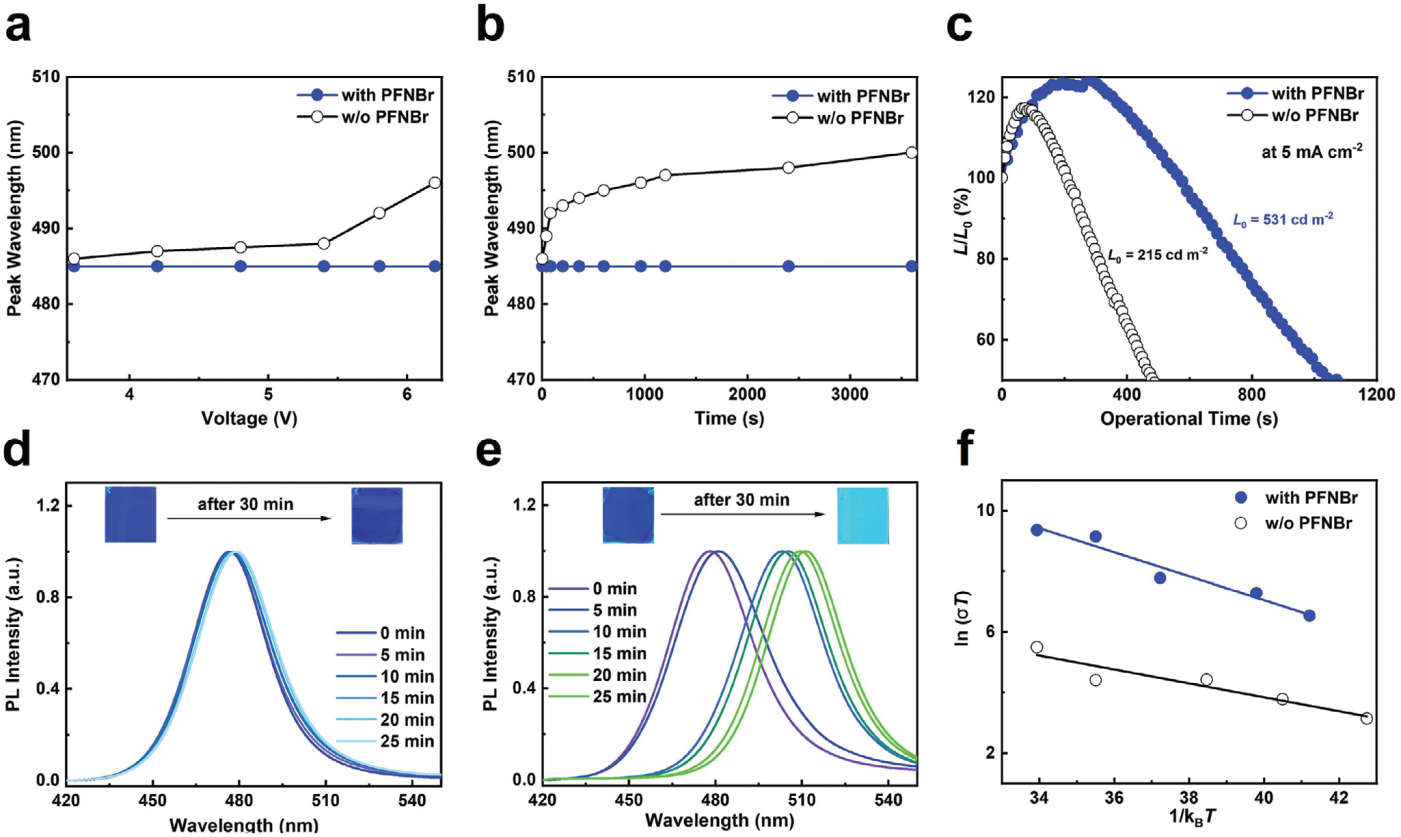
Spectral stability properties of the devices and perovskite films. a) Operating voltage‐dependent changes of the electroluminescence peak wavelength in the sky‐blue perovskite LEDs with (blue) and without (black) PFNBr modification. b) Temporal evolution of the electroluminescence peak wavelength in the sky‐blue perovskite LEDs with and without PFNBr modification operating under a constant voltage of 4.0 V. c) Operational lifetime measurement for the sky‐blue perovskite LEDs with and without PFNBr modification measured under a constant current density of 5 mA cm^−2^. d,e) Time evolution of the PL spectra for the sky‐blue perovskite films with (d) and without (e) PFNBr modification, and the inset shows a photograph of the corresponding films before and after heating at 120 °C for 30 min. f) The temperature‐dependent ion conductivity of the sky‐blue perovskite films with and without PFNBr modification.

In addition, the beneficial effect of PFNBr in the optoelectronic quality of the quasi‐2D perovskite emitter is accompanied by a remarkable improvement of both its color and operational stability. Notably, the EL peak position of the PFNBr‐passivated sky‐blue LED remains unchanged with increasing bias voltage (from 3.0 to 6.2 V) or under continuous bias operation (at 4.0 V for 60 min) (**Figure** **3**a,b). Conversely, as bias voltage increased from 3.0 to 6.2 V, the EL peak position of the control device shifts from 486 to 496 nm. As shown in Figure 3c, the *T*_50_ (time to reach 50% of initial luminance) of sky‐blue device with PFNBr‐containing is 17.5 min under a constant current density of 5 mA cm^−2^, with a twofold increase in operational stability compared to the control device. The trend in the operational stability for true‐ and deep‐blue devices remains the same with the sky‐blue devices (Figure S15, Supporting Information). In order to understand whether the loss of signal is caused by an intrinsic instability of the emitter or not, we monitor the time evolution of the PL spectra for the standalone perovskite films with and without PFNBr modification. As shown in Figure S16, Supporting Information, the PL spectrum of the film without PFNBr modification shifts from 475 to 486 nm, whereas the PL spectrum of the PFNBr‐modified film does not present noticeable changes for more than 1 h under continuous UV light (365 nm) illumination (4 W cm^−2^). We also studied the PL spectral stability of the films under heating at 120 °C for 30 min. As expected, the emission peak of the film without PFNBr modification undergoes a severe bathochromic shift from 475 to 510 nm, while the PFNBr‐modified film shows extremely color‐stable blue emission (Figure 3d,e). These results confirm that PFNBr is indeed able to stabilize the emission spectra of mixed‐halide perovskites. We thus hypothesize that the migration and segregation of halide ions in the perovskite films could be suppressed by the large steric hindrance or the positively charged of the quaternary ammonium ionic group in PFNBr.^[^ ^30^, ^33^, ^34^ ^]^ To further verify our hypothesis, we carried out temperature dependent ion conductivity measurements to estimate the ion migration activation energies (*E*_a_) in the material. As shown in Figure 3f, PFNBr‐modified perovskite film exhibits an *E*_a_ of 396 ± 20 meV, which is over 20% higher than the film without PFNBr modification (321 ± 15 meV). Our approach thus represents a novel strategy to generate metal halide perovskite films with intrinsically higher energetic barriers for ion migration.

**Figure 4:**
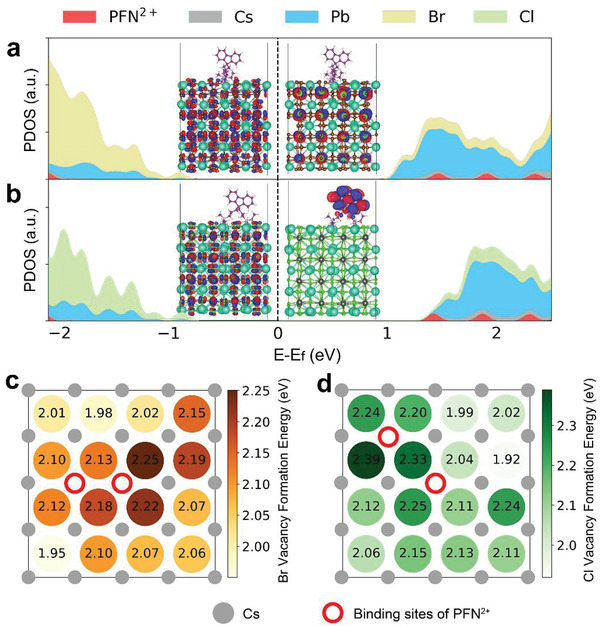
DFT Calculation. The projected density of states (PDOS) of the PFN^2+^ passivated a) CsPbBr_3_ and b) CsPbCl_3_ slab. The Fermi level, *E*_f_, is indicated by the vertical dashed line. The wavefunction of the VBM and CBM states of the passivated slab are shown as blue and red iso‐surfaces in the inset. The top view of the c) CsPbBr_3_ and d) CsPbCl_3_ perovskite with the only top CsBr and CsCl layer is shown. The most stable binding sites of the PFN^2+^ are indicated by the red circle. The difference in vacancy formation energy, *E*(V_halide_^+^), between a passivated and an unpassivated perovskite surface are presented for all the surface halide sites.

To understand the interactions between PFNBr and the perovskite at the atomistic level, we performed density functional theory (DFT) calculations. Perovskites with four atomic layers were created with the top and bottom surfaces being the {100} faces terminated by the CsBr or CsCl layer. The top surfaces contained two Cs^+^ vacancies and were passivated with a model short‐chain π‐conjugated monomer with the same cationic group as PFN^2+^, resulting in trap free surfaces (**Figure** **4**a,b). The binding affinities were 8.05 and 7.61 eV per monomer for CsPbBr_3_ and CsPbCl_3_, respectively (details in Note S1, Supporting Information). The corresponding binding sites are shown in Figure 4c,d. This supports our hypothesis that the PFNBr polymer can bind strongly to the perovskite surfaces and reduce the defect densities of positively charge vacancies. The conduction band minimum (CBM) and valence band maximum (VBM) states of the passivated surfaces for CsPbBr_3_ were delocalized in the perovskite, and for CsPbCl_3_, the VBM is delocalized while the CBM is localized on the PFN^2+^. Our calculations also showed that the surface halide (Br/Cl) formation energy (*E*(V^+^_Br/Cl_)) for passivated perovskite is about 2.1–2.4 eV higher than that for unpassivated surfaces (Figure 4c,d), and the *E*(V^+^_Br/Cl_) is highest near the binding site of the PFN^2+^. We therefore expect lower halide vacancy densities for the passivated surface, minimizing the negative optoelectronic impact caused by halide ion migrations.

**Figure 5:**
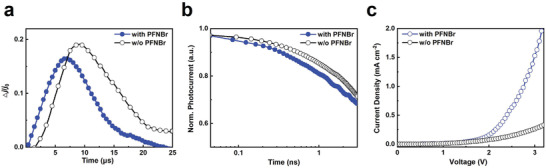
Charge transport properties of the devices. a) CELIV measurements for the sky‐blue perovskite films with and without PFNBr modification. b) Transient photocurrent response recorded from the sky‐blue devices with and without PFNBr modification. c) *J*–*V* curves for the hole‐only devices (ITO/PVK [8 nm]/perovskite with and without PFNBr [15 nm]/TAPC [30 nm]/Al [100 nm]).

To have further inspection on electric properties of modified perovskite, we conducted charge extraction by linearly increasing voltage (CELIV) experiments on basis of our devices. As shown in **Figure** **5**a, the PFNBr‐modified device demonstrates a faster extraction rate, which is related to a higher charge mobility. The charge mobility for the PFNBr‐modified perovskite device is estimated to be 3.95 × 10^−4^ cm^2^ (V s)^−1^, which is 60% higher than that of the film without PFNBr modification (2.44 × 10^−4^ cm^2^ (V s)^−1^) (Figure S17, Supporting Information). We derived the charge transport time constants (τ_t_) from the transient photocurrent decays measured at short circuit in the nanosecond regime. The τ_t_ decreases from 1.4 ns for the device with PFNBr modification to 2.3 ns for the control device, further confirming the enhanced charge transport through PFNBr‐modified perovskite film (Figure 5b). We discard any rearrangement in crystalline orientation in the perovskite film since grazing incidence wide angle X‐ray scattering (GIWAXS) experiments do not reveal changes upon PFNBr incorporation. (Figure S18, Supporting Information). Thus, we speculate that the excellent charge carrier transport properties in our devices are directly associated to the addition of π‐conjugated polymer into the system, which could be attributed to the reduced scattering from defect healing and the intrinsic semiconducting properties of conjugated polymers.^[^ ^39^, ^40^, ^41^ ^]^

Having elucidated the charge transporting properties of PFNBr modified perovskite system, we further investigated the valence band position of perovskite and the highest occupied molecular orbital (HOMO) level of PFNBr by using ultraviolet photoelectron spectrum. As shown in Figure S19, Supporting Information, the valence band and HOMO levels are estimated to be −5.82, −5.60, and −5.34 eV for perovskite without PFNBr modification, PFNBr‐containing perovskite, and PFNBr, respectively. Therefore, the PFNBr‐containing perovskite with shallow HOMO level and PFNBr with π‐conjugated skeleton are beneficial for the hole injection and transport from the perovskite grain boundaries to the bulk. In order to further verify our hypothesis, we investigated the hole‐only devices (HODs) and the electron‐only devices (EODs) with the structure of ITO/PVK (8 nm)/perovskite with and without PFNBr (15 nm)/TAPC (30 nm)/Al (100 nm) and ITO/LiF (3 nm)/perovskite with and without PFNBr (15 nm)/PO‐T2T (30 nm)/LiF (1 nm)/Al (100 nm), respectively. Figure 5c shows the *J*–*V* characteristics of the HODs. The *J* of HOD based on perovskite with PFNBr modification was much higher than that of HOD without PFNBr passivation at the same *V* points, indicating that the hole injection and transport ability of the PFNBr‐modified film is better than that of the film without PFNBr passivation. However, EODs do not show evident differences in the electron transport ability (Figure S20, Supporting Information). This observation indicates that PFNBr mainly boosts the hole transport throughout the perovskite crystallites. The difference between hole and electron transport properties of the device with PFNBr modification is relatively small. Above evidence support the inference that the perovskite LED with PFNBr modification delivers balanced hole and electron transport characteristics.

In addition, we inspected the charge transport properties of the perovskite films with dielectric polymers (polyethylene oxide, poly (2‐hydroxyethyl methacrylate, and poly(2‐(trimethylamino)ethyl methacrylate)) instead of the PFNBr (Figure S21, Supporting Information). The charge transport properties of PFNBr‐modified perovskite films are superior to that of the perovskite films with any dielectric polymer modification. This observation evidences the key differential functionality of the π‐conjugated polymer in the performance of perovskite LEDs, playing an active role in the working mechanism of the system beyond the mere film‐formation control attained by the widely employed insulating polymers.

## 3 Conclusions

In summary, we have introduced an efficient π‐conjugated cationic polymer modification approach for mixed‐halide blue perovskite LEDs with the EL spectra from sky‐blue to deep‐blue region. Our study reveals that PFNBr not only passivates defects and heals grain boundary to enhance the PLQY and stability, but also enables better charge transport properties for the devices. As a result, we demonstrate that wide‐range blue emissive perovskite LEDs with improved efficiency and spectral stability. This novel chemical treatment shows great potential for the identification of strategies to design and realize efficient and stable perovskite‐based LEDs covering the entire visible spectrum.

## 4 Experimental Section

### Materials and Chemicals

PFNBr, CsBr, PbBr_2_, PbCl_2_, DMSO (anhydrous, 99.9%), and chlorobenzene were purchased from Sigma‐Aldrich. PVK (molecular weight 25 000–50 000 g mol^−1^) and PEABr were purchased from Xi'an Polymer Light Technology Corp. PO‐T2T (99.9%) and 8‐hydroxyquinolatolithium (Liq) were purchased from Luminescence Technology. All materials were directly used without further purification.

### Precursor Solution Preparation

For precursor preparation, PEABr, CsBr and (PbCl_2_ + PbBr_2_) with molar ratio at 0.8:1:0.95 were dissolved in DMSO to give an overall concentration of 7 wt%. For PFNBr modification, 0.5 mg mL^−1^ PFNBr was added to the precursor mixture. For emissive spectra covering from sky‐blue to deep‐blue, the molar ratio of PbCl_2_/PbBr_2_ were 1:1 (EL peak 485 nm), 3:2 (EL peak 476 nm), 2:1 (EL peak 468 nm), and 3:1 (EL peak 458 nm). The solution was stirred for 2 h prior to use. PVK was dissolved in chlorobenzene at a concentration of 5 mg mL^−1^.

### Devices Fabrication and Characterization

ITO‐patterned glass substrates were cleaned sequentially with detergent water, acetone, and ethanol for 30 min each, by sonication. Clean ITO substrates were treated with oxygen plasma for 20 min before use. Devices were fabricated with a multilayer structure of ITO (140 nm)/PVK (8 nm)/perovskite (15 nm)/PO‐T2T (25 nm)/Liq (2 nm)/Al (100 nm). The PVK solution was filtered through a 0.2 µm PTFE membrane filter before use. It was spin‐coated onto ITO substrates at 6000 rpm for 35 s, followed by annealing at 120 °C for 15 min. Perovskite films were subsequently deposited onto this PVK layer. The fabrication process was completed by evaporation of PO‐T2T (25 nm), Liq (2 nm), and Al (100 nm) in a thermal evaporation chamber at a vacuum pressure <4 × 10^−4^ Pa. The device had an active area of 0.1 cm^2^. All devices were encapsulated before testing. The cross‐sectional morphologies, EDX mapping, and structures of the full devices were investigated using a HR‐TEM FEI Talos F200X. The samples were prepared by vertical etching using focused ion beam equipment. The current–voltage characteristics were measured with a computer controlled Keithley 2400 source meter. EL spectra were collected by using a Photo Research spectrometer PR650. Devices were swept from zero bias to forward bias. The EQE of the devices (calculated in the range of 380–780 nm) were obtained by measuring the light intensity in the forward direction. All the measurements were carried out in ambient (Figure S22, Supporting Information). Operational lifetime measurement was carried out by using an integrating sphere (Hamamatsu A10094) and a photonic multichannel analyzer PMA‐12 (Hamamatsu C10027‐01).

### Perovskite Films Deposition and Characterization

All perovskite films were deposited by spin‐coating at 6500 rpm for 90 s and then treated at 65 °C for 5 min. GIXRD spectra were obtained by using X‐ray diffractometer with a Cu Kα source (PANalytical B.V. Empyrean). GIWAXS were collected at the I07 Surface and Interface Diffraction beamline at the Diamond Light Source (Didcot, UK). The beam energy was 10 keV (1.23985 Å). The scattered beam was collected by a Pilatus 2M large area detector, at a sample‐detector distance of 400 mm and calibrated with a silver behenate (AgBe) sample. The incidence angle was kept at 0.1° to achieve surface‐preferential probing with a frame exposure of 1 s. The sample chamber was continuously purged with a 1 L min^−1^ He flow. Data was processed with the DAWN software package. FTIR spectroscopy measurements were conducted by using absorption infrared spectrometer (VERTX 70). A field‐emission SEM (Quanta 200 FEG, FEI Co.) was used to acquire SEM images. Atomic force microscopy (AFM) measurements were conducted on a Cypher‐S AFM. Absorption spectra of perovskite films were recorded with a Shimadzu UV2700 spectrometer. Steady‐state PL spectra were collected with a Fluromax 4 fluorescence spectrophotometer. Temperature dependent PL spectra and lifetime measurements were conducted by using Hamamatsu TDC unit M12977 and compact fluorescence lifetime spectrometer C11367; the excitation source was picosecond light pulser C10196 (337 nm). Time‐resolved PL of the perovskite films were measured using an Andor electrically gated intensified charge‐coupled device and laser excitation at 400 nm. PLQY measurements were taken in an integrating sphere and photoexciting with a 405 nm continuous‐wave laser. The laser and the emission signals were measured and quantified using a calibrated Andor iDus DU490A InGaAs detector for the determination of PL quantum efficiency. The external PLQY was calculated according to the previous study.^[^ ^42^ ^]^ Temperature‐dependent measurements were performed by using an Oxford closed‐cycle nitrogen cryostat.

For picosecond transient absorption experiments, the output of a Ti:sapphire amplifier system (Spectra‐Physics Solstice Ace) operating at 1 kHz and generating 80‐fs pulses was split into the pump and probe beam paths. The broadband probe (from ultraviolet to visible regions) was generated using a calcium fluoride crystal. The 400‐nm pump beam was created by sending the 800‐nm fundamental beam of the Solstice Ace through a second harmonic generating beta barium borate crystal of 1 mm thickness (Eksma Optics). The pump was blocked by a chopper wheel rotating at 500 Hz while a computer operated a mechanical delay stage (Newport) to adjust the delay between the pump and the probe. The transmitted pulses were collected with Si and InGaAs dual‐line array detector (Hamamatsu G11608‐512) driven and read out by a custom‐built board from Stresing Entwicklungsbüro.

The capacitance versus frequency plot and Mott–Schottky characterization were carried out by using Solartron ModuLab XM MTS. Trap state distribution (*N*_T_) in energy (*E*_ω_) was deduced on basis of followingequation:^[^ ^43^ ^]^

(1) $$N_{T}\left(E_{\omega }\right)\;\;=\;\;-\frac{V_{bi}}{qW}\;\;\frac{dC}{d\omega }\;\;\frac{\omega }{k_{B}T}$$

(2) $$E_{\omega }\;\;=\;\;k_{B}\;T\left(\ln\frac{2\pi v_{0}T^{2}}{\omega }\right)$$

where *V*_bi_ was the built‐in potential and W was the depletion width, q was the elementary charge, *C* was the capacitance, ω was the applied frequency, *T* was the temperature, *k*_B_ was the Boltzmann constant, *v*_0_ was the characteristic transition frequency. *V*_bi_ and W were obtained from the Mott–Schottky analysis.

The temperature‐dependent conductivity was measured by the following previously established processes.^[^ ^44^ ^]^ On the basis of the Nernst–Einstein relationship:

(3) $$\sigma T\;\;=\;\;\sigma_{0}\exp\left(-E_{a}/k_{B}T\right)$$

where *k*_B_ was Boltzmann's constant, σ_0_ was a constant, σ and *T* were conductivity and temperature, respectively, and *E*_a_ was the activation energy for ion migration. *E*_a_ could be derived from the slope of the ln(*σT*) versus 1/*T* plot.

The CELIV measurement was conducted on ITO (140 nm)/PVK (8 nm)/perovskite (15 nm)/PO‐T2T (25 nm)/Liq (2 nm)/Al (100 nm). A 405 nm laser source modulated by a function generator was used to excite samples, and the triangular wave was implemented on ITO and Al to extract photogenerated carriers. The signal was recorded by an oscilloscope (Agilent 33522A). Carrier mobility (*µ*) were calculated on basis of following equation:^[^ ^45^ ^]^

(4) $$\mu \;\;=\;\;\frac{2d^{2}}{3At_{\max}^{2}\left[1\;+\;0.36\frac{\Delta j}{j_{0}}\right]}$$

where *d* and *A* were the film thickness and the voltage increase rate, *t*_max_ was the time at maximum Δ*j* of the extraction peak, and *j*_0_ was the capacitive displacement current. To compare the charge extraction, the extraction peak Δ*j* was normalized by *j*_0_.

For transient photocurrent measurement, the devices were set in short‐circuit with a 20 Ω resister. A 337 nm nitrogen molecular laser (SRS NL100) was used to excite device, and the signal was recorded by an oscilloscope (TDS3054B, Tektronix).

### Density Functional Theory Calculations

All DFT calculations were performed using Quantum Espresso (v6.6).^[^ ^46^ ^]^ The exchanged correlation was approximated by the PBEsol functional^[^ ^47^ ^]^ and the core–valence interactions were treated using the ultrasoft pseudopotentials from the GBRV library.^[^ ^48^ ^]^ The electronic wavefunctions were expanded in a plane wave basis with a cutoff of 40 Ry and charge‐density cutoff of 200 Ry, and all atomic positions were relaxed until the residual forces were <0.01 eV A^−1^. The perovskites supercell contained 4 × 4 cubic unit cell in the *xy*‐plane with approximate lattice parameter of 24 Å. A vacuum spacing of 15 Å was added to the supercell in the *z*‐direction, and for isolated ligand in all three spatial directions to remove any spurious interactions. Only the Γ‐gamma in the Brillouin zone was sampled. The formation energy correction for a charged defect in 2D slab was computed using a model Gaussian charge within the sxdefectalign2d code,^[^ ^49^ ^]^ and the experimental dielectric constant of 4.8 and 4.5 for CsPbBr_3_ and CsPbCl_3_, respectively.

## Conflict of Interest

The authors declare no conflict of interest.

## Author Contributions

S.Y. and L. S.C. conceived the idea and designed the experiments. L. J.D. conducted the transient absorption experiments and analyzed the data under the supervision of N.C.G. Y.L performed the theoretical calculations. S.Y. and Q.‐W.L. carried out the device fabrication and characterizations under the supervision of Z. K.W. and L. S.L. F.A. and Y. Q.S. commented on the GIXRD results and edited the manuscript. E.R. and M.A. carried out the GISAXS and GIWAXS measurements under the supervision of S.D.S. X.‐P.Z. conducted the HR‐TEM measurements under the supervision of O.M.B. M.‐A.J. participated in the discussion of the photophysical mechanism. Y.‐J.Y. and Y.K.Q. performed low temperature PL measurements. L. S.C., L. S.L., and R.H.F. supervised the work. S. Y. and L. S.C. analyzed all data and wrote the manuscript. All authors edited and commented on the manuscript.

## Supporting information

Supporting Information

## Data Availability

The data that support the findings of this study are available from the corresponding author upon reasonable request.
