# Supplementary material for: Efficient and Spectrally Stable Blue Perovskite Light‐Emitting Diodes Employing a Cationic π‐Conjugated Polymer
Source: Adv Mater. 2021 Sep 24;33(45):2103640. doi: 10.1002/adma.202103640 (PMC11468820; doi:10.1002/adma.202103640)
Supplement: Supplementary file 1 — Supporting Information [file ADMA-33-2103640-s001.pdf]

© 2021 Wiley-VCH GmbH

ADVANCED MATERIALS

Supporting Information

for *Adv. Mater.,* DOI: 10.1002/adma.202103640

Efficient and Spectrally Stable Blue Perovskite Light-Emitting Diodes Employing a Cationic π-Conjugated Polymer

Shuai Yuan, Lin-Song Cui, Linjie Dai, Yun Liu, Qing-Wei Liu, Yu-Qi Sun, Florian Auras, Miguel Anaya, Xiaopeng Zheng, Edoardo Ruggeri, You-Jun Yu, Yang-Kun Qu, Mojtaba Abdi-Jalebi, Osman M. Bakr, Zhao-Kui Wang,* Samuel D. Stranks, Neil C. Greenham, Liang-Sheng Liao,* and Richard H. Friend**

Supporting Information

Efficient and spectrally stable blue perovskite light-emitting diodes employing a cationic π-conjugated polymer

Shuai Yuan #, Lin-Song Cui #★, Linjie Dai, Yun Liu, Qing-Wei Liu, Yu-Qi Sun, Florian Auras, Miguel Anaya, Xiao-Peng Zhen, Edoardo Ruggeri, You-Jun Yu, Yang-Kun Qu, Mojtaba Abdi-Jalebi, Osman M. Bakr, Zhao-Kui Wang★, Samuel D. Stranks, Neil C. Greenham, Liang-Sheng Liao★, Richard H. Friend★

S. Yuan, Q. W. Liu, Y. J. Yu, Y. K. Qu, Prof. Z. K. Wang and Prof. L.S. Liao
Jiangsu Key Laboratory for Carbon-Based Functional Materials & Devices
Institute of Functional Nano & Soft Materials (FUNSOM)
Soochow University
Suzhou 215123, China
E-mail: zkwang@suda.edu.cn; lsliao@suda.edu.cn

S. Yuan, Prof. L. S. Cui
Department of Polymer Science and Engineering
University of Science and Technology of China
Hefei, Anhui 230026, China
E-mail: lscui@ustc.edu.cn

Prof. L. S. Cui, L. J. Dai, Yun. Liu, Y. Q. Sun, F. Auras, M. Anaya, E. Ruggeri, M. A. Jalebi, Prof. S. D. Stranks, Prof. N. C. Greenham and Prof. R. H. Friend
Cavendish Laboratory
University of Cambridge
J.J. Thomson Avenue, Cambridge CB3 0HE, U.K
E-mail: rhf10@cam.ac.uk

X. P. Zheng and Prof. O. M. Bakr
Division of Physical Sciences and Engineering
King Abdullah University of Science and Technology (KAUST)
Thuwal 23955-6900, Kingdom of Saudi Arabia

Keywords: perovskite light-emitting diodes; high efficiency; blue emission; stability; ion migration

Table of Contents

Supplementary Figure S1. Chemical structure and of photophysical characterization of PFNBr. a, Chemical structure of PFNBr. **b,** PL spectra of sky blue perovskite, PL and phosphorescence spectra of PFN-Br, where singlet energy of PFNBr can be determined by room temperature fluorescence as 3.87 eV, and triplet energy of PFNBr is 3.26 eV according to the phosphorescence spectrum measured at 77 K.

Supplementary Figure S2. Tunable emission spectra of Br/Cl mixed perovskite films from sky-blue to deep-blue. a, Photographs of perovskite films under excitation (365 nm) with and without PFNBr, the emission of perovskite films covering sky blue to deep blue from left to right. **b,** the corresponding absorption spectra of perovskite films with PFNBr modification. **c, d** and **e**, the PL spectra of perovskite films with emission wavelength from 470 nm to 451 nm.

Supplementary Figure S3. Angular distribution of radiation intensity for the device with PFNBr modification.

Supplementary Figure S4. Devices' performance for deep blue emissive perovskite films (EL peak: 468 nm) with and without PFNBr modification. **a,** J-V-L curves of devices with and without PFNBr. **b,** The corresponding EQE curves.

Supplementary Figure S5. Devices' performance for deep blue emissive perovskite films (EL peak: 458 nm) with and without PFNBr modification. **a,** J-V-L curves of devices with and without PFNBr. **b,** The corresponding EQE curves.

Supplementary Figure S6. Cross sectional SEM image for devices with PFNBr modification

Supplementary Figure S7. Dynamic light scattering (DLS) cluster size distribution curves for the perovskite precursor solutions with and without PFNBr in DMSO after stirring 2 hours, confirming the formation of different aggregates with and without PFNBr.

Supplementary Figure S8. The zoom-in FTIR spectra of the sky-blue perovskite films without (black) and with PFNBr modification (blue), and the pure PFNBr film (yellow).

Supplementary Figure S9. SEM (upper) and AFM (lower) images of the perovskite film with (a) and without (b) PFNBr modification. The root-mean-square (RMS) roughness values are 0.75 nm and 1.43 nm for the perovskite film with and without PFNBr modification, respectively.

Supplementary Figure S10. TEM images of peeled off 3D perovskite (Br/Cl). a, pristine perovskite film and **b,** PFNBr modified sample, where we increased PFNBr additive content from 0.5mg/ml to 5mg/ml.

Supplementary Figure S11. Integrated PL intensities of the perovskite film with and without PFNBr modification against measuring temperatures. Temperature-dependent PL measurements were conducted to determine the exciton binding energy (E_b). As shown in Figure S11, the E_b of the PFNBr-modified film is estimated to be 141.6 meV, which is much higher than that of the film without PFNBr modification (98.7 meV). Such a large E_b is beneficial to confine the excitons in the nanograins for efficient radiative recombination.

Supplementary Figure S12. Transient absorption contour maps of the PFNBr-modified perovskite film on nanosecond timescales.

In order to gain insights into the energy transfer processes in the blue quasi-2D perovskites, transient absorption (TA) spectroscopy was performed for the PFNBr-modified perovskite film. As shown in **Figure 2b** and **Supplementary Figure S10**, three distinctive ground state bleach (GSB) peaks at 414 nm, 445 nm and 456 nm are observed. According to the second derivative of the steady state absorption in **Figure 2a**, the first two sharp bleaches agree well with the quasi-2D perovskite absorption features of the bilayer (n = 2) and trilayer (n = 3) phases, whereas the third broad bleach feature is attributed to the spectrally overlapped signals of the $n \geq 4$ components. The bandgap of these quasi-3D components with $n \geq 4$ can be determined by the boundary between the band edge bleach feature and the bandgap renormalization feature at the early times (0.1 ps). The latter appears at 474 nm for our perovskites, as shown in **Figure 2b**, and this bandgap is related to the emissive species. **Figure 2c** shows the dynamic evolution of the three bleach bands (414 nm, 445 nm and 456 nm) and the band edge of the emissive spices (474 nm). At time zero, the three bleach bands build up very fast and simultaneously, indicating that the initial photoexcitations are formed

in all three domains under 400 nm excitation. Following the fast buildup, the bilayer (n = 2) shows a dominant ultrafast decay with a time constant of 0.43 ps, corresponding to the depopulation of n = 2 component. This agrees well with a second-stage fast buildup of the other two bleach bands. After fast charge delocalization from the quasi-2D (n = 2, 3) to quasi-3D domains, charge carriers within the quasi-3D domains undergo fast cooling processes to the band edge with a time constant of approximately 1 ps, confirmed by the buildup of the 474 nm bleach and the recovery of the bandgap renormalization signal. The cooling process is also accompanied by a small red-shift of the large-n bleach peak (**Figure 2b**). Since excitons are more tightly bound in quasi-2D perovskites, they are less susceptible to local defects. These bound electron-hole pairs can be concentrated effectively towards the quasi-3D domains on sub-ps timescales, leading to an efficient energy migration process.

Supplementary Figure S13. Excitation-intensity-dependent PLQY of the perovskite films with and without PFNBr modification; the PLQY of the perovskite film with PFNBr modification is as high as 82 % under the excitation intensity of 0.2 mW cm^{-2}.

Supplementary Figure S14. The capacitance behaviour of the devices. a, Capacitance versus frequency plot for the devices with and without PFNBr modification. **b,** Mott–Schottky curves for the devices with and without PFNBr modification.

Supplementary Figure S15. Driven voltage dependent EL spectra for deep blue perovskite LEDs with and without PFNBr modification.

Supplementary Figure S16. PL aging test under 365 nm light illumination (4 W cm^{-2}). a, Normalized PL spectra of the perovskite film with PFNBr modification. **b,** Normalized PL spectra of the pristine perovskite film.

Supplementary Figure S17. Charge extraction by linearly increasing voltage (CELIV) measurement for perovskite films with and without PFNBr modification.

Supplementary Figure S18. GIWAXS pattern of perovskite films with (a) and without (b) PFNBr modification.

Supplementary Figure S19. UPS result for PFNBr modified perovskite film.

Supplementary Figure S20. ***J-V*** **curves for the electron-only devices (ITO/ LiF (3 nm)/ Perovskite with and without PFNBr (15 nm)/ PO-T2T (30 nm)/ LiF(1 nm)/ Al (100 nm)).**

Supplementary Figure S21. ***J-V*** **curves for the perovskite LED devices with various perovskite films, where PFNBr and three typical linear polymers (PEO, poly-HEMA and poly-MAEM) were incorporated. Higher injection current in the device with PFNBr modification confirm the better carriers transporting property in corresponding perovskite film (ITO/ PVK (8 nm)/ Perovskite with and without Polymer (15 nm)/ PO-T2T (25nm)/ LiF(1 nm)/ Al (100 nm)).**

Supplementary Figure S22. LED device characterization system. *J-V* characteristics were driven and collected by Keithley 2400, while PhotoReasearch Spectrometer PR650 was used for light output measurements.

Supplementary Table S1. Comparison of our blue perovskite LEDs with other reported blue perovskite LEDs.

Supplementary Table S2. Turn-on voltages for all the relevant devices.

Supplementary Table S3. Estimated the grain size for quasi-2D and 3D phases.

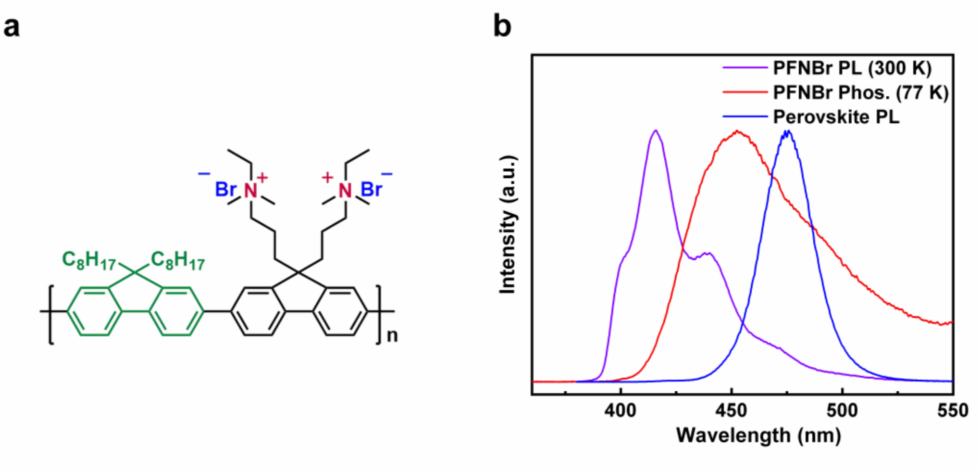

Supplementary Figure S1. Chemical structure and of photophysical characterization of PFNBr. a, Chemical structure of PFNBr. **b,** PL spectra of sky blue perovskite, PL and phosphorescence spectra of PFN-Br, where singlet energy of PFNBr can be determined by room temperature fluorescence as 3.87 eV, and triplet energy of PFNBr is 3.26 eV according to the phosphorescence spectrum measured at 77 K.

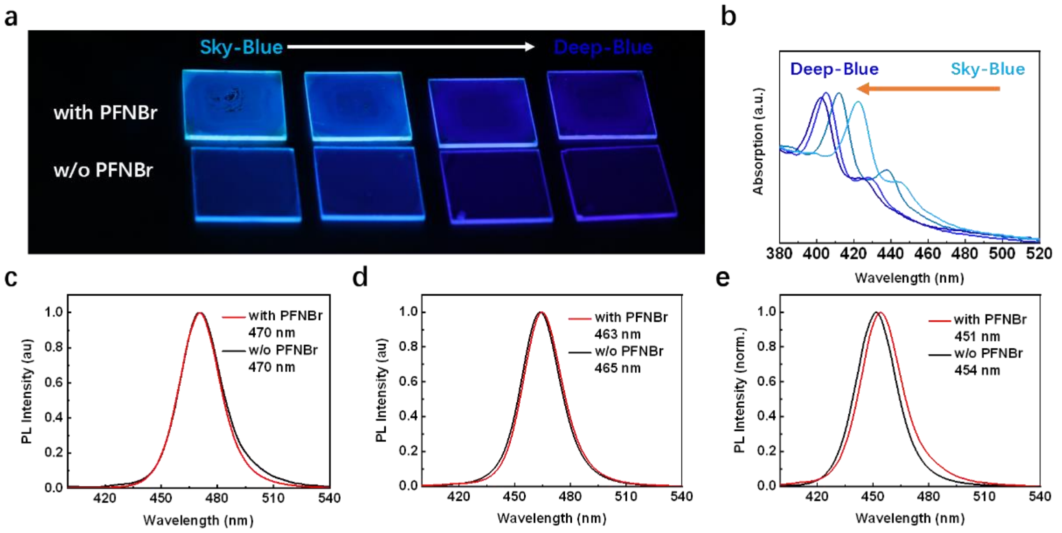

Supplementary Figure S2. Tunable emission spectra of Br/Cl mixed perovskite films from sky-blue to deep-blue. a, Photographs of perovskite films under excitation (365 nm) with and without PFNBr, the emission of perovskite films covering sky blue to deep blue from left to right. **b,** the corresponding absorption spectra of perovskite films with PFNBr modification. **c, d** and **e**, the PL spectra of perovskite films with emission wavelength from 470 nm to 451 nm. The slightly excess Br^- ion from PFNBr increases halide ratio (Br/Cl) in the final products leading to slightly lower optical bandgap in modified perovskite films.

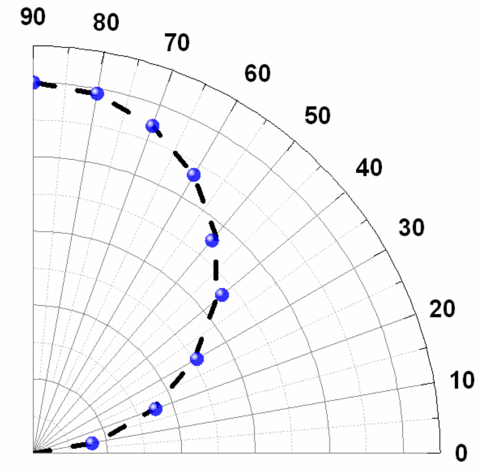

Supplementary Figure S3. Angular distribution of radiation intensity for the device with PFNBr modification.

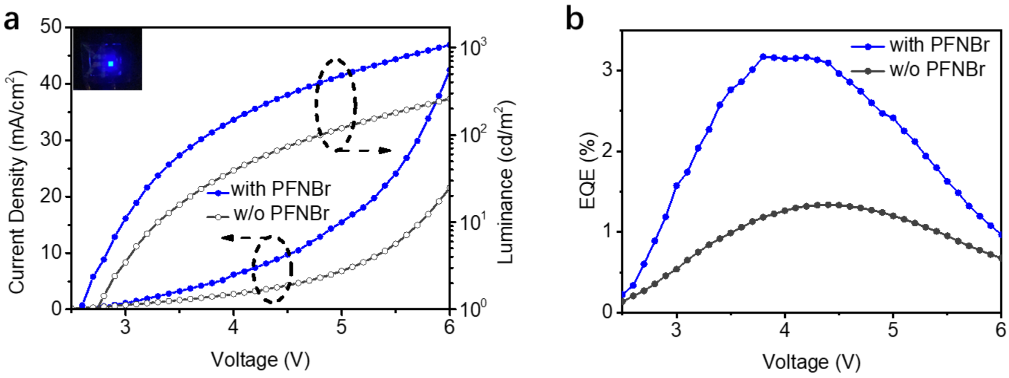

Supplementary Figure S4. Devices' performance for deep blue emissive perovskite films (EL peak: 468 nm) with and without PFNBr modification. **a,** J-V-L curves of devices with and without PFNBr. **b,** The corresponding EQE curves.

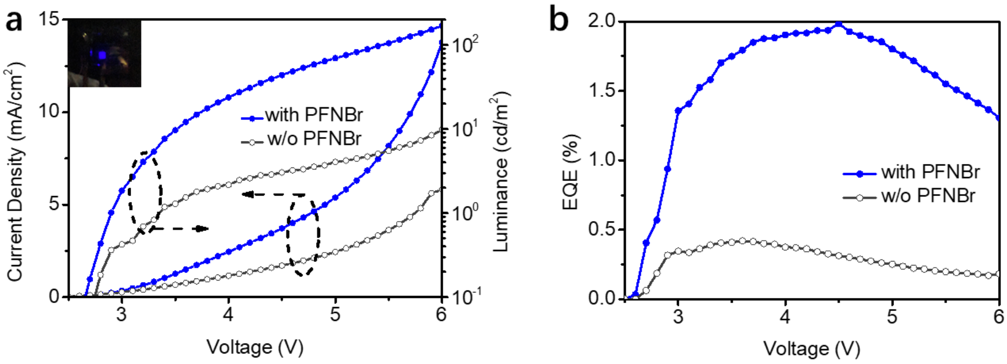

Supplementary Figure S5. Devices performance for deep blue emissive perovskite films (EL peak: 458 nm) with and without PFNBr modification. **a,** J-V-L curves of devices with and without PFNBr. **b,** The corresponding EQE curves.

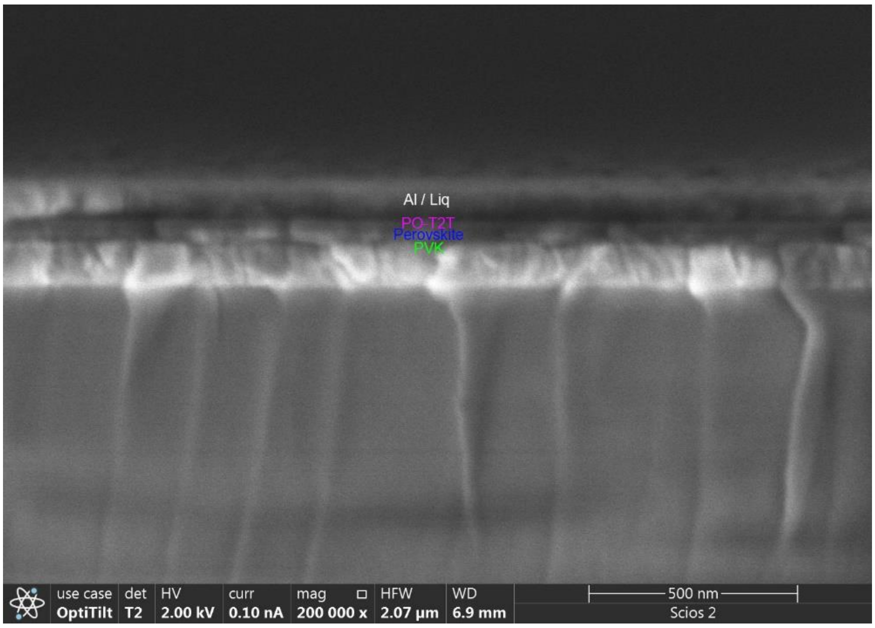

Supplementary Figure S6. Cross sectional SEM image for devices with PFNBr modification

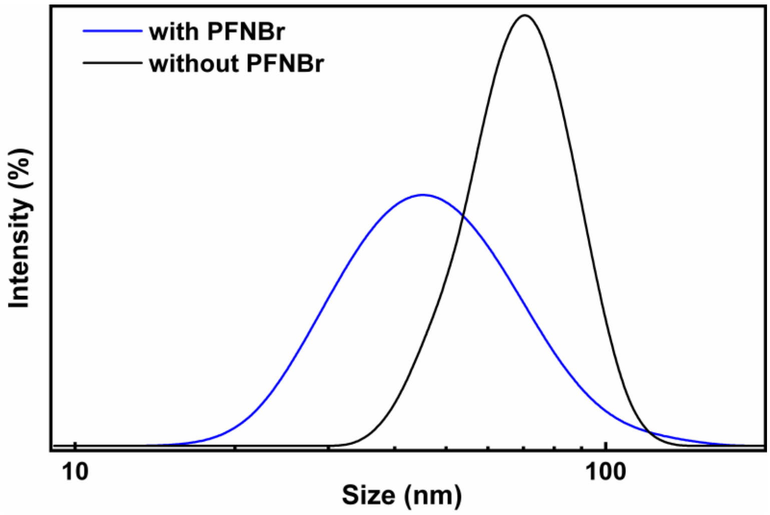

Supplementary Figure S7. Dynamic light scattering (DLS) cluster size distribution curves for the perovskite precursor solutions with and without PFNBr in DMSO after stirring 2 hours, confirming the formation of different aggregates with and without PFNBr.

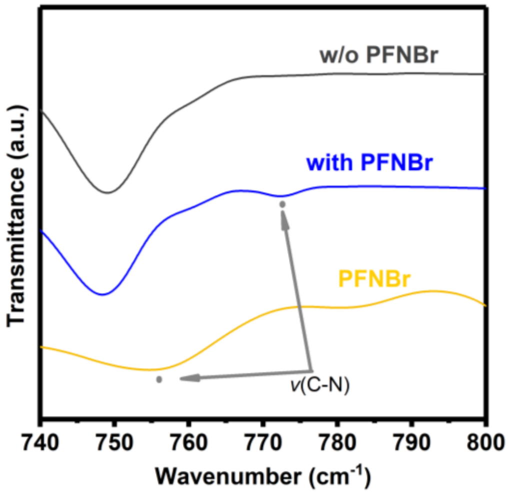

Supplementary Figure S8. The zoom-in FTIR spectra of the sky-blue perovskite films without (black) and with PFNBr modification (blue), and the pure PFNBr film (yellow).

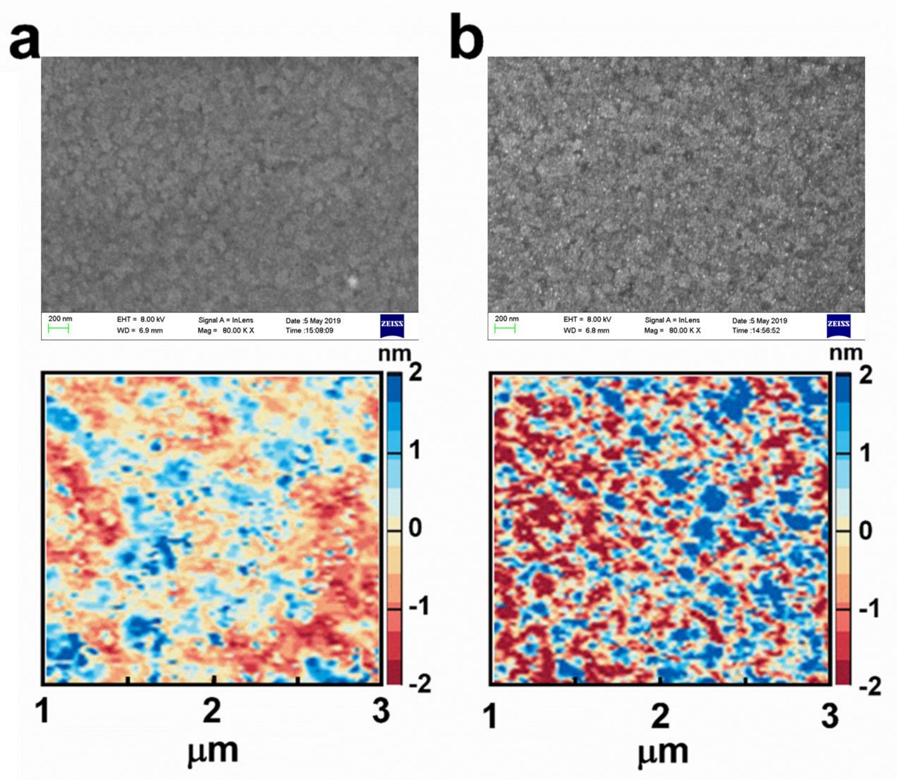

Supplementary Figure S9. SEM (upper) and AFM (lower) images of the perovskite film with (a) and without (b) PFNBr modification. The root-mean-square (RMS) roughness values are 0.75 nm and 1.43 nm for the perovskite film with and without PFNBr modification, respectively.

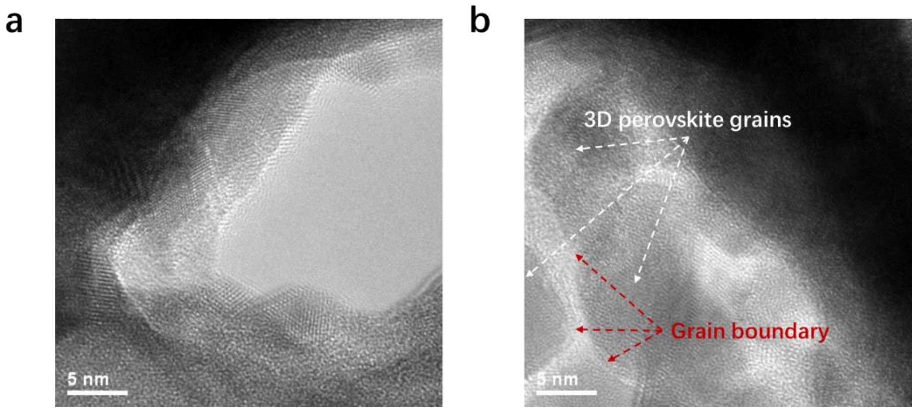

Supplementary Figure S10. TEM images of peeled off 3D perovskite (Br/Cl). a, pristine perovskite film and **b,** PFNBr modified sample, where we increased PFNBr additive content from 0.5mg/ml to 5mg/ml.

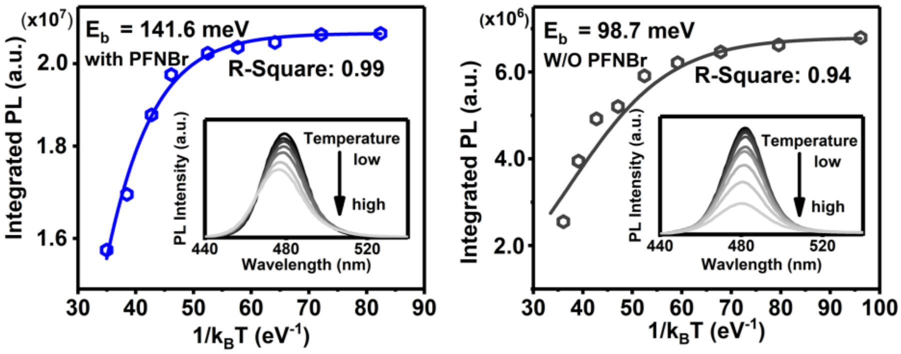

Supplementary Figure S11. Integrated PL intensities of the perovskite film with and without PFNBr modification against measuring temperatures. Temperature-dependent PL measurements were conducted to determine the exciton binding energy (E_b). As shown in Figure S11, the E_b of the PFNBr-modified film is estimated to be 141.6 meV, which is much higher than that of the film without PFNBr modification (98.7 meV). Such a large E_b is beneficial to confine the excitons in the nanograins for efficient radiative recombination.

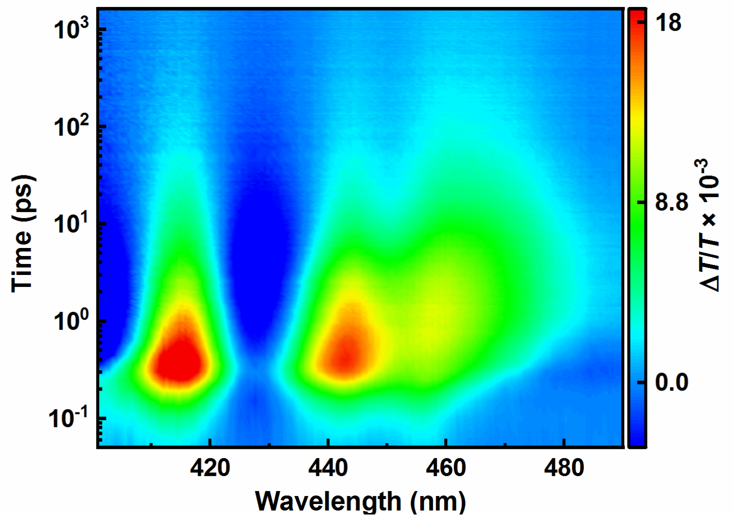

Supplementary Figure S12. Transient absorption contour maps of the PFNBr-modified perovskite film on nanosecond timescales.

In order to gain insights into the energy transfer processes in the blue quasi-2D perovskites, transient absorption (TA) spectroscopy was performed for the PFNBr-modified perovskite film. As shown in **Figure 2b** and **Supplementary Figure S10**, three distinctive ground state bleach (GSB) peaks at 414 nm, 445 nm and 456 nm are observed. According to the second derivative of the steady state absorption in **Figure 2a**, the first two sharp bleaches agree well with the quasi-2D perovskite absorption features of the bilayer (n = 2) and trilayer (n = 3) phases, whereas the third broad bleach feature is attributed to the spectrally overlapped signals of the $n \geq 4$ components. The bandgap of these quasi-3D components with $n \geq 4$ can be determined by the boundary between the band edge bleach feature and the bandgap renormalization feature at the early times (0.1 ps). The latter appears at 474 nm for our perovskites, as shown in **Figure 2b**, and this bandgap is related to the emissive species. **Figure 2c** shows the dynamic evolution of the three bleach bands (414 nm, 445 nm and 456 nm) and the band edge of the emissive spices (474 nm). At time zero, the three bleach bands build up very fast and simultaneously, indicating that the initial photoexcitations are formed in all three domains under 400 nm excitation. Following the fast buildup, the bilayer (n = 2) shows a dominant ultrafast decay with a time constant of 0.43 ps, corresponding to the depopulation of n = 2 component. This agrees well with a second-stage fast buildup of the other two bleach bands. After fast charge delocalization from the quasi-2D (n = 2, 3) to quasi-3D domains, charge carriers within the quasi-3D domains undergo fast cooling processes to the band edge with a time constant of approximately 1 ps, confirmed by the buildup of the 474 nm bleach and the recovery of the bandgap renormalization signal. The cooling process is also accompanied by a small red-shift of the large-n bleach peak (**Figure 2b**). Since excitons

are more tightly bound in quasi-2D perovskites, they are less susceptible to local defects. These bound electron-hole pairs can be concentrated effectively towards the quasi-3D domains on sub-ps timescales, leading to an efficient energy migration process.

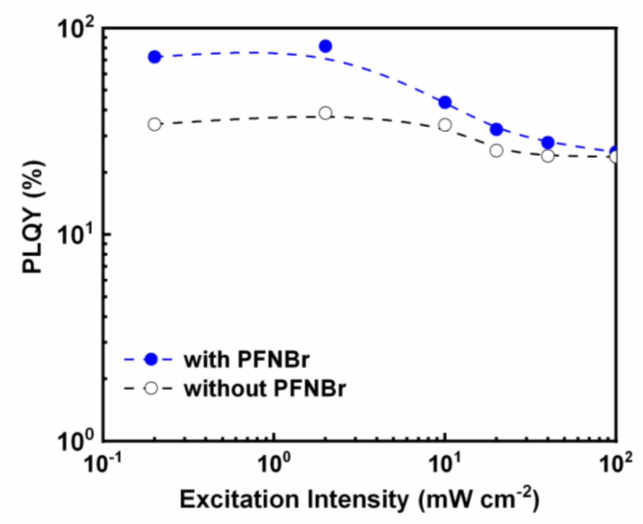

Supplementary Figure S13. Excitation-intensity-dependent PLQY of the perovskite films with and without PFNBr modification; the PLQY of the perovskite film with PFNBr modification is as high as 82 % under the excitation intensity of 0.2 mW cm^{-2}.

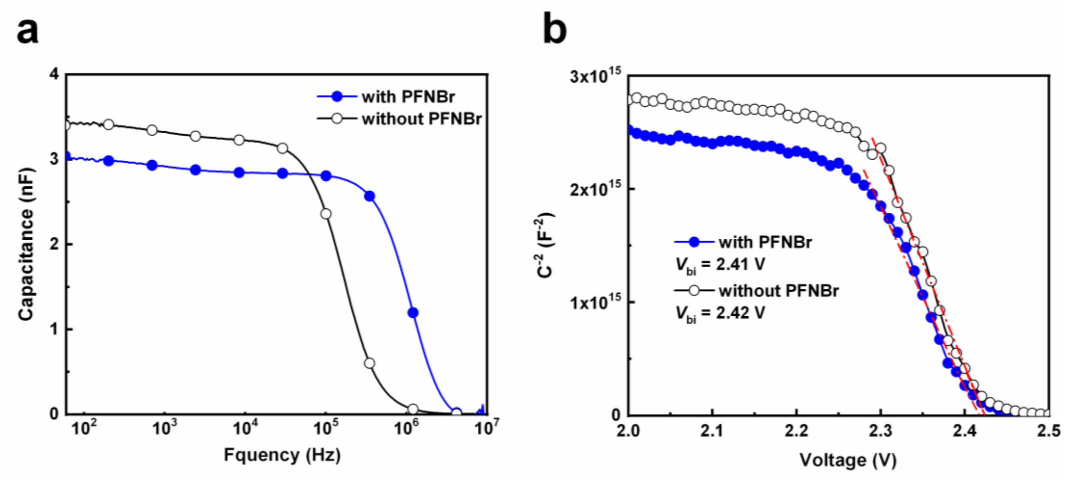

Supplementary Figure S14. The capacitance behaviour of the devices. a, Capacitance versus frequency plot for the devices with and without PFNBr modification. **b,** Mott–Schottky curves for the devices with and without PFNBr modification.

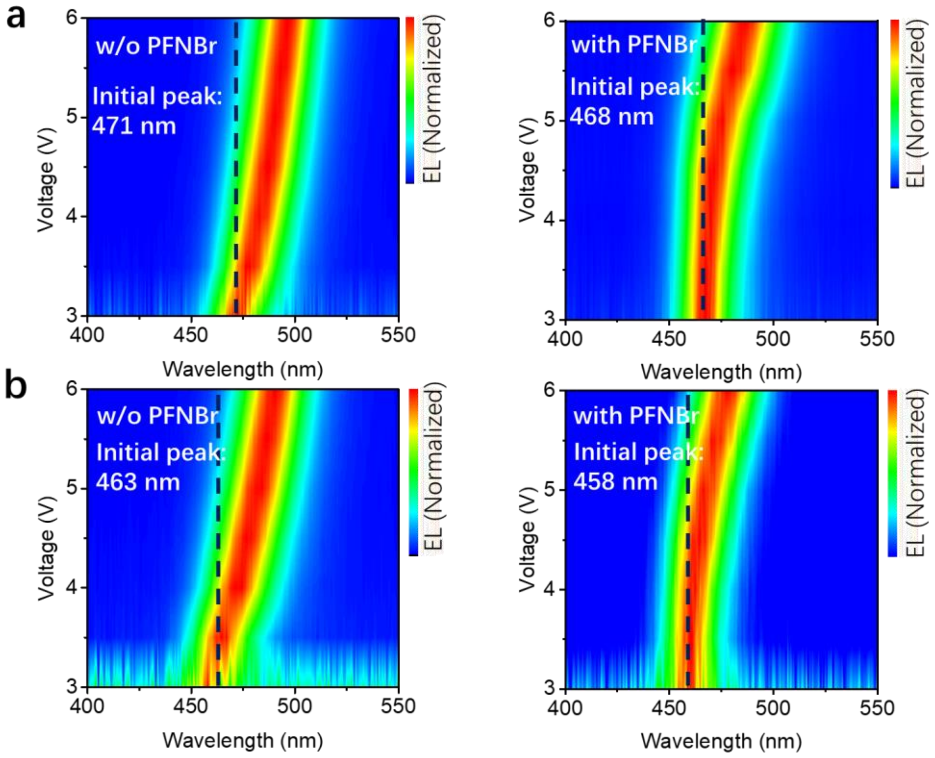

Supplementary Figure S15. Driven voltage dependent EL spectra for deep blue perovskite LEDs with and without PFNBr modification.

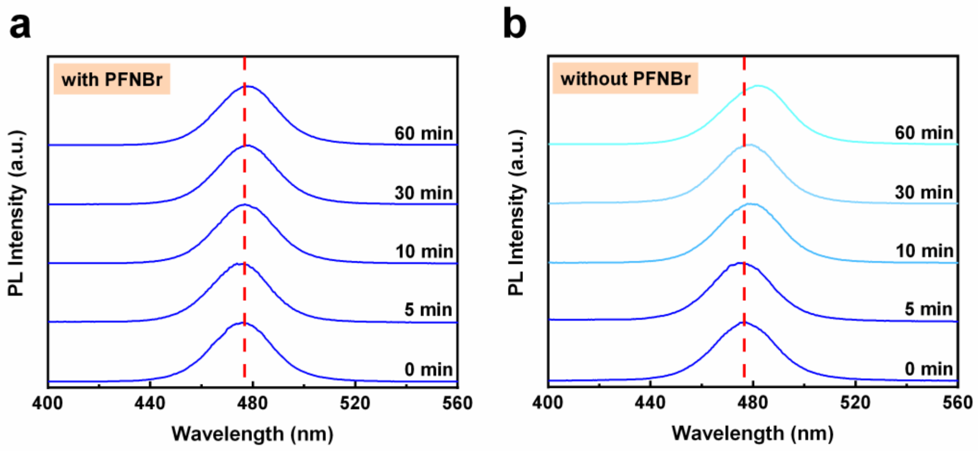

Supplementary Figure S16. PL aging test under 365 nm light illumination (4 W cm^{-2}). a, Normalized PL spectra of the perovskite film with PFNBr modification. **b,** Normalized PL spectra of the pristine perovskite film.

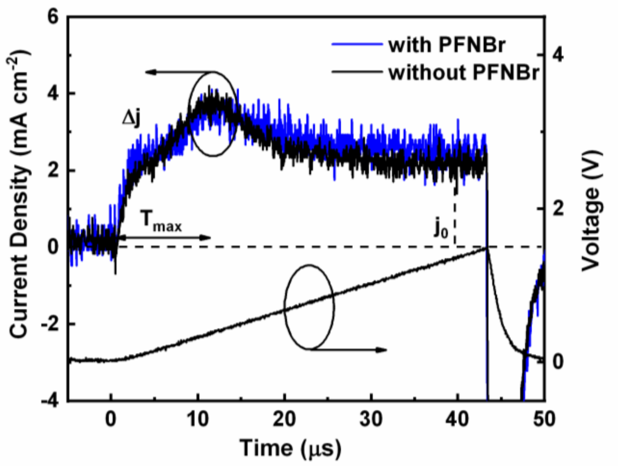

Supplementary Figure S17. Charge extraction by linearly increasing voltage (CELIV) measurement for perovskite films with and without PFNBr modification.

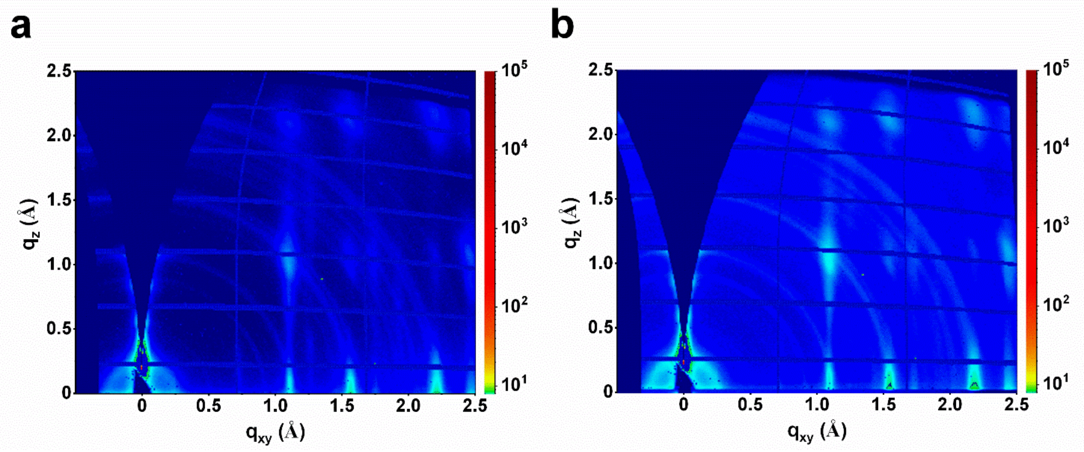

Supplementary Figure S18. GIWAXS pattern of perovskite films with (a) and without (b) PFNBr modification.

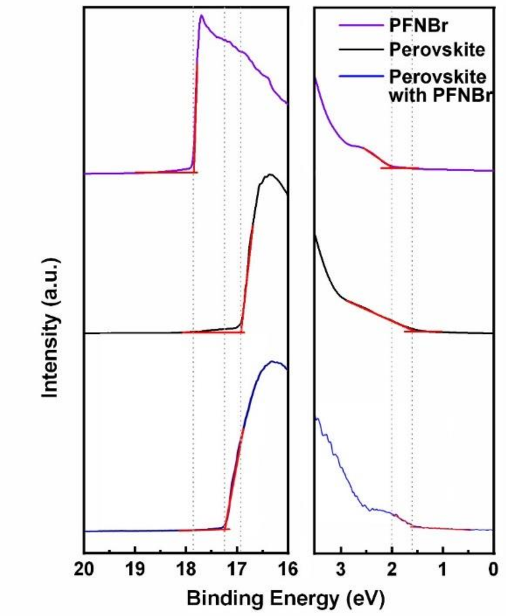

Supplementary Figure S19. UPS result for PFNBr modified perovskite film.

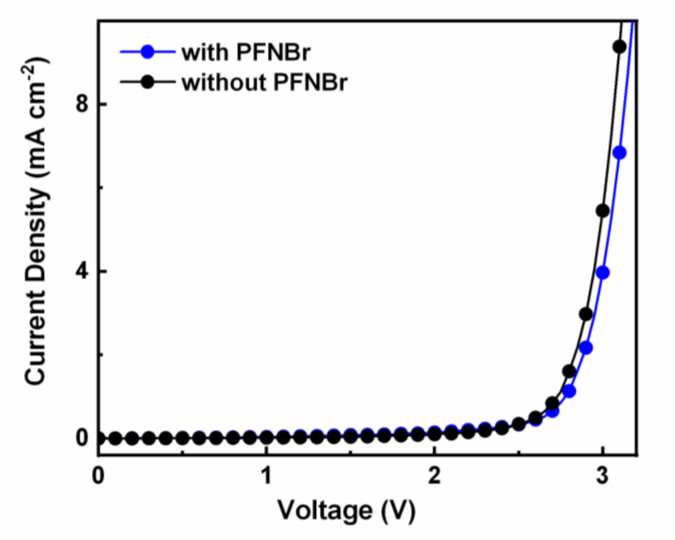

Supplementary Figure S20. ***J-V*** **curves for the electron-only devices (ITO/ LiF (3 nm)/ Perovskite with and without PFNBr (15 nm)/ PO-T2T (30 nm)/ LiF(1 nm)/ Al (100 nm)).**

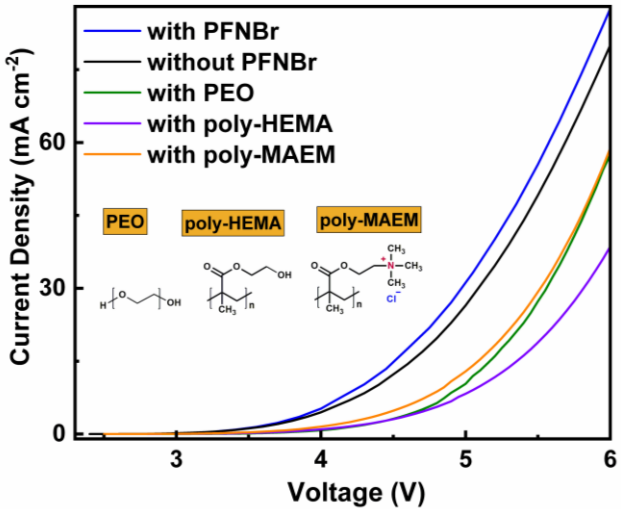

Supplementary Figure S21. ***J*-*V* curves for the perovskite LED devices with various perovskite films, where PFNBr and three typical linear polymers (PEO, poly-HEMA and poly-MAEM) were incorporated. Higher injection current in the device with PFNBr modification confirm the better carriers transporting property in corresponding perovskite film (ITO/ PVK (8 nm)/ Perovskite with and without Polymer (15 nm)/ PO-T2T (25nm)/ LiF(1 nm)/ Al (100 nm)).**

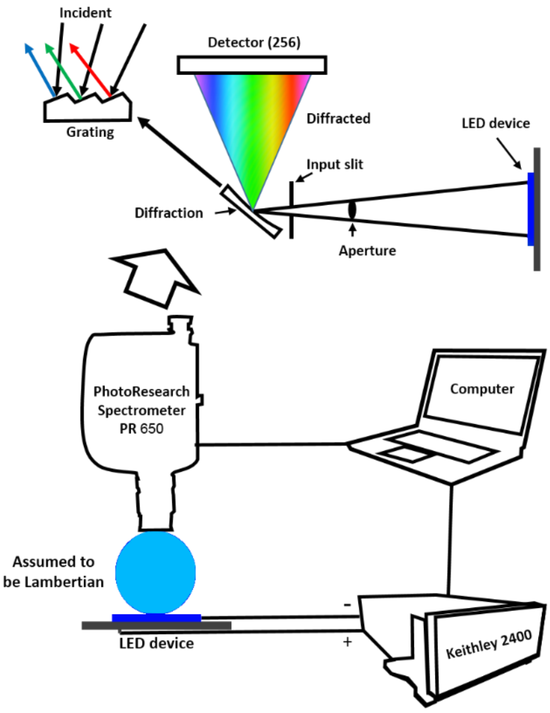

Supplementary Figure S22. LED device characterization system. *J*-*V* characteristics were driven and collected by Keithley 2400, while PhotoReasearch Spectrometer PR650 was used for light output measurements.

Supplementary Table S1. Comparison of our blue perovskite LEDs with other reported blue perovskite LEDs.

EL Peak (nm)	EQE (%)	Reference
**485**	**11.2**	**This Work**
**476**	**8.0**	
**467**	**3.2**	
**458**	**1.9**	
483	9.5	*Nat. Photon.* **2019**, 13, 760
474	4.0	
485	11.0	*Nat. Commun.* **2019**, 10, 5633
477	4.8	
488	12.1	*Nat. Commun.* **2020**, 11, 4165
480	4.1	
480	5.7	*Nat. Commun.* **2019**, 10, 1027
478	2.8	
490	1.5	*Nat. Commun.* ***2018**, 9, 3541*
476	1.3	*Nat. Commun.* ***2019**, 10, 1868*
473	8.8	*Nat. Commun.* ***2020**, 11, 6428*
479	12.3	*Nat. Nanotech.* ***2020**, 15, 668*
477	11.0	*Nat. Commun.* ***2021**, 12, 361*

Supplementary Table S2. Turn-on voltages for all the relevant devices.

Perovskite based LEDs<br>Emission Peak (nm)		Turn-on voltage (V)
485 nm	w/o PFNBr	2.62
	With PFNBr	2.55
476 nm	w/o PFNBr	2.46
	With PFNBr	2.48
468 nm	w/o PFNBr	2.75
	With PFNBr	2.61
458 nm	w/o PFNBr	3.3
	With PFNBr	2.9

The slightly lower turn-on voltages for devices with emission at 476 nm can be attributed to the fabrication processes such as spin-coating process, thermal annealing process and evaporation process, meanwhile, the measurement error such as delay time after each driven current point, it may be impacted by transient capacitance, also probably induces the slight difference.

Supplementary Table S3. Estimated the grain size for quasi-2D and 3D phases.

Thin film	Diffract Peak (2θ)	FWHM (rad)	Grain Size (nm)
Perovskite without PFNBr modification	5.37 /2D (002) n=1	0.0050	30.8
Perovskite without PFNBr modification	4.04 /2D (002) n=2	0.0032	48.1
Perovskite without PFNBr modification	15.96/3D (100)	0.0115	13.5
Perovskite with PFNBr modification	5.34 /2D (002) n=1	0.0065	23.7
Perovskite with PFNBr modification	4.01 /2D (002) n=2	0.0089	17.3
Perovskite with PFNBr modification	15.89/3D (100)	0.0195	7.9

Supplementary Note 1

The binding affinity (E_{bind}) of the cationic polymer to the perovskite surface is computed as

$$E_{\mathrm{bind}} = E_{\mathrm{tot}} - E_{\mathrm{slab}} - E_{\mathrm{ligand}}$$

where E_{tot} is the total energy of the passivated perovskite supercell, E_{slab} is the energy of the perovskite slab with a pair of Cs vacancy, and E_{ligand} the energy of the ligand.

The formation energy of the halide vacancy $E^f[V_{halide}^+]$ for a perovskite slab is computed[1] as

$$E^f[V_{\mathrm{halide}}^+] = E_{\mathrm{tot}}[V_{\mathrm{halide}}^+] - E_{\mathrm{tot}}[\mathrm{bulk}] + \mu_{\mathrm{halide}} + E_F + E_{\mathrm{corr}}$$

Where $E_{tot}[V_{halide}^+]$ is the total energy of the supercell containing the halide vacancy, E_{tot}[bulk] is the total energy for the equivalent supercell without the defect. μ_{halide} is the chemical potential for the halide atom which is the same for the passivated and unpassivated slab. E_F is the Fermi level of the electron referenced to the VBM. *E*corr is the correction term for the charged defect calculation in the periodic 2D supercell, which were computed using sxdefectalign2D code[2].

Reference

[1] C. Freysoldt, B. Grabowski, T. Hickel, J. Neugebauer, G. Kresse, A. Janotti, C. G. Van de Walle, *Rev. Mod. Phys.* **2014**, 86, 253.

[2] C. Freysoldt, J. Neugebauer, *Physical Review B* **2018**, 97, 205425.
